# Supplementary material for: Numerical magnitude, rather than individual bias, explains spatial numerical association in newborn chicks
Source: eLife. 2020 Jun 25;9:e54662. doi: 10.7554/eLife.54662 (PMC7316507; doi:10.7554/eLife.54662)
Supplement: Transparent reporting form [file elife-54662-transrepform.pdf]

eLIFE
1st Floor
24 Hills Road
Cambridge CB2 1JP, UK
P 01223 855340
W elifesciences.org
T @elife

eLife's transparent reporting form

We encourage authors to provide detailed information *within their submission* to facilitate the interpretation and replication of experiments. Authors can upload supporting documentation to indicate the use of appropriate reporting guidelines for health-related research (see EQUATOR Network), life science research (see the BioSharing Information Resource), or the ARRIVE guidelines for reporting work involving animal research. Where applicable, authors should refer to any relevant reporting standards documents in this form.

If you have any questions, please consult our Journal Policies and/or contact us: editorial@elifesciences.org.

Sample-size estimation

- You should state whether an appropriate sample size was computed when the study was being designed
- You should state the statistical method of sample size computation and any required assumptions
- If no explicit power analysis was used, you should describe how you decided what sample (replicate) size (number) to use

Please outline where this information can be found within the submission (e.g., sections or figure legends), or explain why this information doesn't apply to your submission:

This information can be found in "Experimental procedure" session.

Replicates

- You should report how often each experiment was performed
- You should include a definition of biological versus technical replication
- The data obtained should be provided and sufficient information should be provided to indicate the number of independent biological and/or technical replicates
- If you encountered any outliers, you should describe how these were handled
- Criteria for exclusion/inclusion of data should be clearly stated
- High-throughput sequence data should be uploaded before submission, with a private link for reviewers provided (these are available from both GEO and ArrayExpress)

Please outline where this information can be found within the submission (e.g., sections or figure legends), or explain why this information doesn't apply to your submission:

- *How often each experiment was performed* can be found in “Material and methods” and in the “Table 2”.
- *A definition of biological versus technical replication.* This is not applicable to our study.
- *The data obtained should be provided and sufficient information should be provided to indicate the number of independent biological and/or technical replicates.* All data and Codes have been uploaded as “source data”.
- “If you encountered any outliers, you should describe how these were handled”: Outliers were included in the final sample and plotted on out graphs, as described in the legend of the Figure 1.
- *Criteria for exclusion/inclusion of data should be clearly stated:* This information can be found in “Material and Methods”, subsections “Shaping and training” and “Test”
- *High-throughput sequence data should be uploaded before submission, with a private link for reviewers provided (these are available from both GEO and ArrayExpress).* This is not applicable to our study

Statistical reporting

- Statistical analysis methods should be described and justified
- Raw data should be presented in figures whenever informative to do so (typically when N per group is less than 10)
- For each experiment, you should identify the statistical tests used, exact values of N, definitions of center, methods of multiple test correction, and dispersion and precision measures (e.g., mean, median, SD, SEM, confidence intervals; and, for the major substantive results, a measure of effect size (e.g., Pearson's r, Cohen's d)
- Report exact p-values wherever possible alongside the summary statistics and 95% confidence intervals. These should be reported for all key questions and not only when the p-value is less than 0.05.

Please outline where this information can be found within the submission (e.g., sections or figure legends), or explain why this information doesn't apply to your submission:

- *Statistical analysis methods should be described and justified*. This information can be found in the following sessions: "Introduction " and "Results".
- *Raw data should be presented in figures whenever informative to do so (typically when N per group is less than 10).* Our sample size is larger than 10.
- *For each experiment, you should identify the statistical tests used, exact values of N, definitions of center, methods of multiple test correction, and dispersion and precision measures (e.g., mean, median, SD, SEM, confidence intervals; and, for the major substantive results, a measure of effect size (e.g., Pearson's r, Cohen's d).* This information is reported in "Results" section.
- *Report exact p-values wherever possible alongside the summary statistics and 95% confidence intervals. These should be reported for all key questions and not only when the p-value is less than 0.05.* This information is reported in "Results" section.

(For large datasets, or papers with a very large number of statistical tests, you may upload a single table file with tests, Ns, etc., with reference to sections in the manuscript.)

Group allocation

- Indicate how samples were allocated into experimental groups (in the case of clinical studies, please specify allocation to treatment method); if randomization was used, please also state if restricted randomization was applied
- Indicate if masking was used during group allocation, data collection and/or data analysis

Please outline where this information can be found within the submission (e.g., sections or figure legends), or explain why this information doesn't apply to your submission:

- *Indicate how samples were allocated into experimental groups (in the case of clinical studies, please specify allocation to treatment method); if randomization was used, please also state if restricted randomization was applied. This* information is reported in "Introduction" and in "Results" sections.
- *Indicate if masking was used during group allocation, data collection and/or data analysis.* This is not applicable to our study

Additional data files ("source data")

- We encourage you to upload relevant additional data files, such as numerical data that are represented as a graph in a figure, or as a summary table
- Where provided, these should be in the most useful format, and they can be uploaded as "Source data" files linked to a main figure or table

- Include model definition files including the full list of parameters used
- Include code used for data analysis (e.g., R, MatLab)
- Avoid stating that data files are "available upon request"

Please indicate the figures or tables for which source data files have been provided:

All data and Codes have been uploaded as "source data". The R codes used for data analyses, including the full list of parameters used have also been uploaded.
Our datasets
Data are stored and backed up on the Research Data Unipd server. The Research Data Unipd is Research Data Repository that meets the demands of a FAIR data storage (Findable, Accessible, Interoperable, Reusable) – in accordance with the Guidelines on FAIR Data Management in Horizon 2020- July 2016.
http://researchdata.cab.unipd.it/id/eprint/326
